# Computational Modeling of Drug Dissolution in the Human Stomach

**DOI:** 10.3389/fphys.2021.755997

**Published:** 2022-01-10

**Authors:** Jung Hee Seo, Rajat Mittal

**Affiliations:** Department of Mechanical Engineering, Johns Hopkins University, Baltimore, MD, United States

**Keywords:** gastric flow, pill tracking, gastrointestinal tract, immersed boundary method, fluid-structure interaction

## Abstract

A computational model of drug dissolution in the human stomach is developed to investigate the interaction between gastric flow and orally administrated drug in the form of a solid tablet. The stomach model is derived from the anatomical imaging data and the motion and dissolution of the drug in the stomach are modeled via fluid-structure interaction combined with mass transport simulations. The effects of gastric motility and the associated fluid dynamics on the dissolution characteristics are investigated. Two different pill densities are considered to study the effects of the gastric flow as well as the gravitational force on the motion of the pill. The average mass transfer coefficient and the spatial distributions of the dissolved drug concentration are analyzed in detail. The results show that the retropulsive jet and recirculating flow in the antrum generated by the antral contraction wave play an important role in the motion of the pill as well as the transport and mixing of the dissolved drug concentration. It is also found that the gastric flow can increase the dissolution mass flux, especially when there is substantial relative motion between the gastric flow and the pill.

## Introduction

The oral route is used most frequently for drug administration in humans due to its safety, reduced cost, and high degree of patient compliance, but it is also the most complex way for an active pharmaceutical ingredient (API) to enter the body. This complexity is because drug absorption via the gastrointestinal (GI) tract depends not only on factors related to the drug and its formulation, but also on the contents of the stomach and stomach motility and the associated fluid dynamics. Administration of oral dosage forms with food in particular, has the potential to affect drug bioavailability due to the dynamic physiological environment of the fed stomach (Spiller, 1986; Koziolek et al., 2016). Pressure and shear forces induced by stomach contractions and buoyancy effects can generate complex pill trajectories and varying rates of dissolution and non-uniform emptying of the drug into the duodenum. In the case of modified-release dosage forms, this can even lead to premature drug release or “gastric dumping” (Weitschies et al., 2005; Koziolek et al., 2016). These issues pose several challenges to the design of drug delivery systems from the perspective of research and development as well as clinical, and regulatory aspects.

The current approach to assessing/quantifying drug dissolution relies primarily on in-vitro models, but recapitulating the conditions experienced by an oral drug formulation in the stomach has been extremely challenging. The USP (United States Pharmacopeia) dissolution apparatus (I-IV) are the de-facto standard (particularly, USP-II : Paddle) in this arena, but a variety of studies have shown significant shortcomings of these devices for mimicking the conditions of the stomach (Abrahamsson et al., 2005; Wang and Armenante, 2016; Gao, 2017). More advanced in-vitro models (Kostewicz et al., 2014; Minekus, 2015) have attempted to mitigate these shortcomings, but at the cost of increasing device complexity. Furthermore, despite this increased complexity, these in-vitro simulators are still unable to adequately recreate biorelevant conditions of gastric motility-induced fluid flow, mixing, shear and pressure forces, and the biochemical status associated with food contents and gastric secretions (Gao, 2017; Butler et al., 2019).

*In-silico* models of drug dissolution in biomimetic models of the human stomach have the potential to overcome many of the above-mentioned limitations of in-vitro models, and these in-silico research could revolutionize our understanding of oral drug delivery systems. So far, in-silico models have been employed for the microscale drug design [(Haddish-Berhane et al., 2007; Mehta et al., 2019), for example] and the drug dissolution in the testing devices (Abrahamsson et al., 2005; Bai and Armenante, 2009), but the biomechanical model of the GI tract has not been considered in those studies. There have been limited but insightful in-silico modeling of GI tract biomechanics and digestion processes (Pal et al., 2004; Ferrua and Singh, 2010, 2011; Ferrua et al., 2011; Trusov et al., 2016; Ishida et al., 2019), but those previous computational studies of gastric function are mostly focused on the mixing and emptying of liquid gastric contents. In order to model the drug dissolution in the stomach, not only does one have to model the flow of gastric contents due to stomach motility, but one also needs to resolve the six degree-of-freedom motion of the pill/tablet in the gastric flow field. Furthermore, modeling of both diffusive and convective transport of the API concentration is essential in order to gain insights into the process of pill dissolution.

In the present study, a computational model of drug dissolution in the physiological human stomach is developed by including the aforementioned features. A human stomach model is derived from the anatomical imaging data, and the pill motion and its dissolution in the stomach are modeled by the fully coupled fluid-structure interaction and mass transport simulations. The present study focuses on the initial dissolution of a non-disintegrating pill to investigate the effects of gastric motility and associated fluid dynamics on the dissolution characteristics. The density of the pill is also an important parameter in the design of oral drug delivery, especially for control of the pill residence time in the GI system (Gupta et al., 2009). In order to study the interaction between the pill and the gastric fluid motion as well as the gravitational force, two different pill densities (specific gravities 1.0 and 1.2) are considered. The overall dissolution rate and the local API concentration distributions are analyzed in detail. The correlation between the fluid shear force on the pill and the surface diffusion rate is also examined to study the effect of gastric fluid flow on the surface erosion of the pill further.

## Materials and Methods

### Human Stomach Model

**FIGURE 1:**
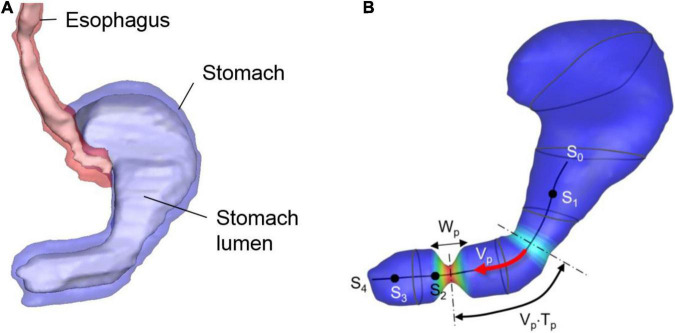
Human stomach model. **(A)** GI tract model segmented from the Virtual Population imaging data. Stomach lumen represents the inner wall of the stomach. **(B)** Stomach lumen model used for the simulations and parameters for the wall motility prescription. See the text for the detail.

A human stomach model is generated by using the “Virtual Population” model (Gosselin et al., 2014) – which has detailed, high-resolution anatomical full body models derived from magnetic resonance image data. The 3D model of the stomach is created by segmenting the stomach lumen from the Virtual Population 1.3 “Duke” model of this dataset (Figure 1A). The segmentation is done by using the software, Materialize MIMICS, and the smoothing and surface mesh generation are done by using the Materialize 3-matic.

The stomach wall motility, especially the antral contraction wave (ACW) is modeled following the previous study (Ferrua et al., 2011). The ACW wall kinematics are modeled as a pulse-wave propagating down toward the pylorus with its axis along the centerline of the antrum (see Figure 1B). The traveling cosine wave of the contraction strain, *λ_*a*_* is modeled by


(1) $$\lambda_{a}\,\left(t,s\right)=\lambda_{a,\max}\,\sum_{n_{p}}\frac{1}{2}\,\left\{\cos\left(2\,\pi \,\frac{s-V_{p}\,{\text{t-n}}_{p}\,T_{p}\,V_{p}}{W_{p}}\right)+1\right\}\,h\,\left(s\right),$$

where *s* is the distance along the antrum centerline, *n*_*p*_ is the pulse count, *V*_*p*_ is the pulse propagation speed, *T*_*p*_ is the pulse interval, *W*_*p*_ is the pulse width, and *h*(*s*) is a spatial amplitude modulation function. In the present study, the parameter values are set to *V_*p*_* = 2.3 mm/sec, *T_*p*_* = 20 sec, *W_*p*_* = 20 mm, and *λ_*a*,_*_*max*_ = 0.7 based on the published data (Ferrua et al., 2011). The centerline is divided into 4 segments as shown in Figure 1B; *s*_0_–*s*_1_: no contraction, *s*_1_–*s*_2_: the ACW amplitude grows, *s*_2_–*s*_3_: a constant amplitude ACW, and *s*_3_–*s*_4_: the ACW diminishes, and this is controlled by the amplitude modulation function, *h*(*s*) given by


(2) $$h\,\left(s\right)=\left\{ \begin{array}{cc} 0 & s<s_{1}\\ \left\{1-\cos\left(\pi \,\left(s-s_{1}\right)/\left(s_{2}-s_{1}\right)\right)\right\}/2 & s_{1}\leq s<s_{2}\\ 1 & s_{2}\leq s\leq s_{3}\\ \left\{1+\cos\left(\pi \,\left(s-s_{3}\right)/\left(s_{4}-s_{3}\right)\right)\right\}/2 & s_{3}<s\leq s_{4} \end{array}\right..$$

The lumen wall contraction is then prescribed by


(3) $${\vec{x}}_{w}={\vec{x}}_{w,0}+\lambda_{a}\,{\vec{r}}_{w},$$

where ${\vec{x}}_{w}$ is the position vector of the lumen wall, ${\vec{x}}_{w,0}$ is the lumen wall position at the initial, undeformed state, and ${\vec{r}}_{w}$ is the vector from the wall to the antrum centerline. Eqs. (1)–(3) generate peristaltic, traveling antral contraction wave, and the wall motility can be controlled by adjusting the model parameters. Note that there could be the substantial variability in the stomach size and shape as well as wall motility, which would affect the gastric flow strength and pattern.

### Flow Solver

**FIGURE 2:**
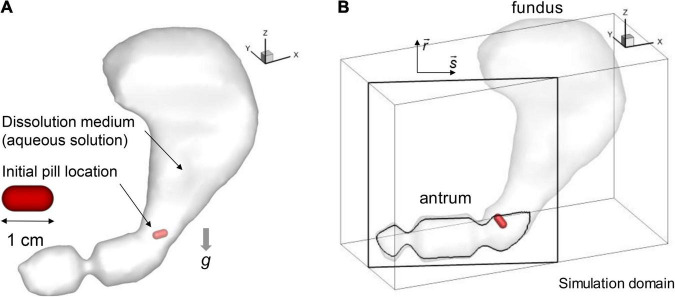
Simulation models. **(A)** Stomach and pill models. **(B)** Simulation domain and the antrum cross-sectional plane (*s*-*r*).

The stomach model described in Section “Human stomach model” is immersed into a Cartesian volume grid to perform the fluid-structure interaction simulation using an in-house immersed boundary method (Mittal et al., 2008). The Cartesian domain (see Figure 2B) size used are 18 cm in x-, 8 cm in y-, and 12 cm in z-direction. This volume is discretized into a total of 360 × 160 × 240 (about 14 million) uniform Cartesian cells with a grid spacing of 0.5 mm. This resolution is based on the grid refinement study for the present model (see Appendix A).

In the current study, we focus on modeling drug dissolution accompanied by an aqueous solution whose properties are those of water. The equations governing the flow of the fluid are the Navier-Stokes equations:


(4) $$\nabla \cdot \vec{u}=0;\rho \,\left(\frac{\partial \vec{u}}{\partial t}+\vec{u}\cdot \nabla \vec{u}\right)+\nabla p=\mu \,\nabla^{2}\vec{u}+\rho \,\vec{g},$$

where $\vec{u}$ is the flow velocity, *p* is the pressure, ρ is the density of the fluid, μ is the viscosity, and $\vec{g}=-G\,\hat{z}$ is the gravitational acceleration vector and *G* = 9.8 m/s^2^. The direction of gravity is determined from the orientation of the imaging data assuming a standing position. The incompressible Navier-Stokes equations (Eq. 4) are advanced in time using a fractional-step based algorithm (Chorin, 1969). A hybrid second-order upwind and central differencing is used for the convection term, and a second-order central-scheme is used for the diffusion terms. Time-integration is performed implicitly with the second-order Crank-Nicolson method and the non-linear convection term is updated iteratively. The pressure Poisson equation solver employs an efficient, immersed boundary based stabilized Bi-conjugate gradient (BiCGSTAB) method (Zhu et al., 2017).

The boundary conditions on the immersed boundary is applied using the sharp-interface immersed boundary method (Mittal et al., 2008). In this method, the surface of the immersed body is represented by an unstructured mesh, which consists of triangular elements. Using the surface mesh, the Cartesian cells in the fluid and solid regions are identified, and the flow equations are solved only in the fluid region. In the present immersed boundary method, no additional forcing term is used to apply the boundary conditions. The boundary conditions on the fluid-solid interface are imposed by prescribing the variable values on the nearest Cartesian cell in the solid region. The details about the present flow solver can be found in the multiple references (Mittal et al., 2008; Seo and Mittal, 2011; Zhu et al., 2017). This flow solver has been extensively validated for a variety of laminar/turbulent flows (Mittal et al., 2008), biological (Zheng et al., 2013a,b) and cardiovascular flows (Vedula et al., 2014), and fluid-structure interaction problems (Bhardwaj and Mittal, 2012; Shoele and Mittal, 2014), and employed for a wide range of studies of biomedical fluid flows (Seo and Mittal, 2013; Seo et al., 2014, 2016; Vedula et al., 2015, 2016; Zhu et al., 2018; Bailoor et al., 2021).

The time step size used in the simulation is Δ*t* = 0.002 s, which resolves one ACW period (*T*_*p*_) with 10,000 time points. Assuming a fully closed pylorus, no-slip and no-penetration boundary condition are applied on all stomach wall except the top part of the fundus. An open boundary condition with zero-gradient pressure and velocity, which allows small amount of inflow and outflow, is applied on the top part of the fundus to satisfy the mass conservation, and to model the accommodation of the fundus.

### Dynamical Model of the Pill

The shape of the pill is modeled as a cylinder with spherical cap ends (see Figure 2A). The length and diameter are set to 1 cm and 0.5 cm, respectively. For the composition, the pill is modeled as a non-disintegrating pill made of salicylic acid following the previous study (Bai and Armenante, 2009). In the present study, two different pill densities (*ρ_*p*_*) are considered for the specific gravities, SG = *ρ_*p*_*/ρ = 1.0 and 1.2. The pill motion in the stomach interacting with the fluid flow is simulated by solving a six-degrees of freedom (6DOF) model of Newton’s Second Law for its linear and angular motions. The governing equations are given by


(5) $$m\,\frac{\partial \vec{v}}{\partial t}={\vec{F}}_{f}+{\vec{F}}_{c}+m\,\vec{g},I\,\frac{\partial \vec{\omega }}{\partial t}={\vec{M}}_{f}+{\vec{M}}_{c},$$

where *m* is the mass and **I** is the moment-of-inertia of the pill, *v* and ω are translational and angular velocities, and *F*_*f*_ and *M*_*f*_ are the force and moment induced by the shear and pressure of the surrounding fluid, and *F*_*c*_ and *M*_*c*_ are ones by contact with the stomach wall. The fluid force and moment are computed by


(6) $${\vec{F}}_{f}=\int_{S_{P}}-p\,\vec{n}+\vec{\tau }\,d\,S,{\vec{M}}_{f}=\int_{S_{P}}\vec{r}\times \left(-p\,\vec{n}+\vec{\tau }\right)\,dS,$$

where *S*_*p*_ is the surface of the pill, τ is the viscous shear stress, and *r* is the vector from the center-of-mass of the pill to the point on the surface.

**FIGURE 3:**
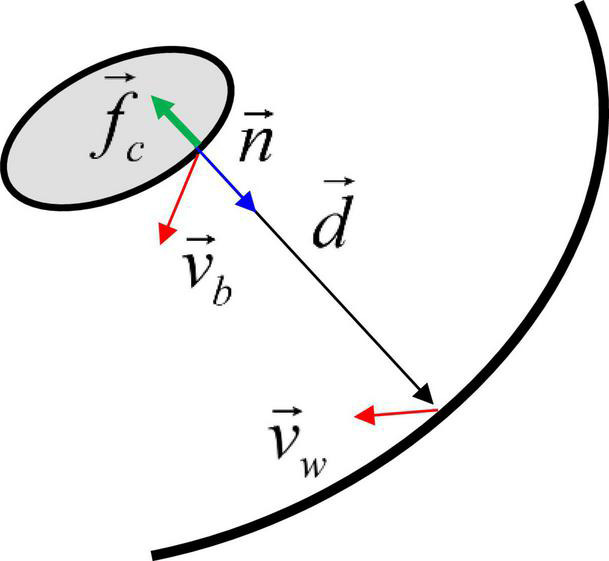
Schematic for the contact force model between the moving body and the wall. See the text for details.


For modeling contact between the pill and the stomach lumen, we employ a non-linear spring-based model. A schematic is shown in Figure 3. First, at each point on the pill surface, the contact point on the stomach wall is determined by finding the nearest point on the wall. Then the distance vector $\vec{d}$ is obtained, and the non-linear spring and damping force are calculated by using this distance vector:

(7) $$\begin{array}{c} \delta =\left|\vec{d}\right|,\dot{\delta }=\left({\vec{v}}_{b}-{\vec{v}}_{w}\right)\cdot \vec{d}/\left|\vec{d}\right|,f_{b}\,\left(\delta \right)=\exp\left(-{\left(\delta /\delta_{\min}\right)}^{4}\right)\\ {\vec{f}}_{c}=\left\{ \begin{array}{cc} -\left(k_{\max}+k_{d}\,\dot{\delta }\right)\,f_{b}\,\vec{d}/\left|\vec{d}\right| & \text{for}\,\vec{n}\cdot \vec{d}>0\\ 0 & \text{otherwise} \end{array}\right. \end{array},$$

where *v*_*b*_ is the pill surface velocity, *v*_*w*_ is the wall velocity, *f*_*b*_ is a non-linear function, δ_*min*_ is the minimum distance parameter, *k*_*max*_ is the spring constant, *k*_*d*_ is the damping constant, and *f*_*c*_ is the contact stress. δ_*min*_ controls the distance range of the contact force activation. Since *f*_*b*_ decays very rapidly for δ > δ_*min*_, the contact force is only active very near the wall, i.e., δ ≤ δ_*min*_. In this study, δ_*min*_ is set to 1.5 mm. Finally, the contact force and moment are obtained by


(8) $${\vec{F}}_{c}=\int_{S_{P}}{\vec{f}}_{c}\,dS,{\vec{M}}_{c}=\int_{S_{P}}\vec{r}\times {\vec{f}}_{c}\,dS.$$

The parameters for the present contact model are *k*_*max*_, *k*_*d*_ and δ_*min*_. *k*_*max*_ represents the maximum contact stress, and to satisfy the no-penetration condition, a scaling analysis can show


(9) $$k_{\max}=C_{k}\,\Delta \,\rho \,g\,L_{p},$$

where Δρ is the density difference between the body and the surrounding fluid, *L*_*p*_ is the length scale of the body which can be obtained by dividing the volume by the frontal area of the body, and *C*_*k*_ is an O(1) constant. In the present study, *C*_*k*_ is set to 2.5. The damping constant needs to be set to suppress unphysical rebound after the contact, and this can be represented by using a relaxation time scale, *t*_*R*_,


(10) $$k_{d}=\rho_{p}\,L_{p}/t_{R},t_{R}=C_{d}\,\Delta \,t.$$

In this study, we set the relaxation time scale relative to the simulation time step size using the constant, *C*_*d*_, which is set to 5 in the current simulations.

### Drug Dissolution

The release of API from the pill and the transport of the API by the flow are simulated by solving the convection and diffusion equation for the mass transport:


(11) $$\frac{\partial C_{A\,P\,I}}{\partial t}+\left(\vec{u}\cdot \nabla \right)\,C_{A\,P\,I}=D\,\nabla^{2}C_{A\,P\,I},$$

where *C*_*API*_ is the concentration of the API and *D* is the mass diffusivity. For the salicylic acid pill, the Schmidt number for the mass diffusion, ν/*D* is about 400 (Bai and Armenante, 2009), where ν is the kinematic viscosity of the water. The dissolution of the drug is modeled by a surface erosion, and the mass transfer rate is given by


(12) $$\frac{d\,m_{d}}{d\,t}=\int_{S_{P}}-{D\,\frac{\partial C_{A\,P\,I}}{\partial n}|}_{w\,a\,l\,l}\,d\,S,$$

where *m*_*d*_ is the dissolved mass and *n* is the surface normal direction. The boundary condition for Eq. (11) on the pill surface is *C_*API*_ = C_*s*_*, where *C*_*s*_ is the surface concentration or the saturated concentration, given by the solubility of the pill. For the salicylic acid, the solubility constant, *ρ_*p*_*/*C*_*s*_ is about 400 (Bai and Armenante, 2009). The estimated surface erosion velocity due to mass transfer is about 2.5 × 10^–6^ cm/s, and thus this effect is neglected. A zero mass-flux condition is applied on the stomach wall. Equation (11) is discretized by using a second-order finite difference scheme in space and advanced in time by a four-stage Runge-Kutta method. The same sharp interface immersed boundary method with the flow solver is also used for the mass transport equation.

### Fluid-Structure Interaction

Since the density ratio of the solid (pill) to the fluid medium (water) is close to 1, the coupled fluid and solid equations (Eqs. 4 and 5) are solved with an implicit scheme. The implicit coupling is done iteratively as follows:


(13) $$\left\{ \begin{array}{c} \left({\vec{u}}^{k+1},p^{k+1}\right)=q\,\left({\vec{x}}^{k},{\vec{v}}^{k},{\vec{u}}^{k},p^{k}\right)\\ {\vec{F}}^{k+1}=f\,\left({\vec{x}}^{k},{\vec{v}}^{k},{\vec{u}}^{k+1},p^{k+1}\right)\\ \frac{{\vec{v}}^{k+1}-{\vec{v}}^{n}}{\Delta \,t}=\vec{g}+\frac{1}{M}\,\left[\alpha \,{\vec{F}}^{k+1}+\left(1-\alpha \right)\,{\vec{F}}^{k}\right]\\ \frac{{\vec{x}}^{k+1}-{\vec{x}}^{n}}{\Delta \,t}={\vec{v}}^{k+1} \end{array}\right.,$$

where the function *q* represents the Navier-Stokes equations (Eq. 4), the function *f* is for the calculation of the forces (Eqs. 6–8), *k* is the iteration index, α is the relaxation parameter, and ${\vec{x}}^{k}$ and ${\vec{v}}^{k}$ are the position and velocity of the solid, respectively. In the iteration process, the flow velocity and pressure (${\vec{u}}^{k+1},p^{k+1}$) are updated first by solving the Navier-Stokes equations. The forces on the solid body (${\vec{F}}^{k+1}$) is then calculated using the updated flow variables. Finally, the velocity and position of the solid body (${\vec{v}}^{k+1},{\vec{x}}^{k+1}$) are updated using this force. For the stability and better convergence, we employed under-relaxation during the solid velocity update. The iteration continues till $\left|{\vec{x}}^{k+1}-{\vec{x}}^{k}\right|<\varepsilon$ and then the solutions at *k*+1 are taken for the next physical time step *n*+1. In this study, we used ε = 0.0001 mm and α = 0.5.

## Results

### Gastric Flow Patterns

**FIGURE 4:**
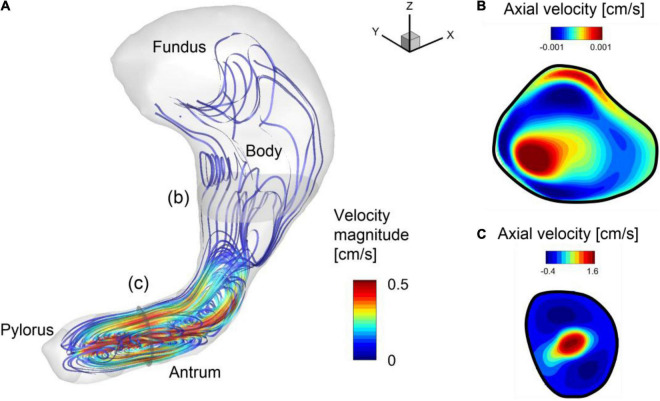
Gastric flow pattern averaged over one antral contraction cycle. **(A)** Streamlines colored by the velocity magnitude. **(B)** Velocity contours on the cross-section in the body (b). Positive velocity is toward the fundus. **(C)** Velocity contours on the cross section in the antrum (c). Positive velocity is toward the body.

The simulation is first performed without the pill to investigate the flow patterns in the stomach. The gastric flow patterns are Eulerian time averaged over one antral contraction cycle (*T_*p*_* = 20 s) and shown in Figure 4. Figure 4A shows the streamlines of the time-averaged gastric flows. One can see the energetic flows consist of jet and recirculation in the antrum region, while the flows in the body and fundus are very weak. This is because the gastric flow is primarily driven by the antral contraction wave. The flow velocity contours on the cross-section in the antrum region are plotted in Figure 4C. It shows a strong jet at the center of the antrum, which is surrounded by a flow toward the pylorus. The jet at the center of the antrum is called the “retropulsive jet,” and has been reported in many previous studies (Pal et al., 2004; Ferrua and Singh, 2010; Ferrua et al., 2011; Trusov et al., 2016) as one of the characteristics of gastric flow during digestion. The strength of the retropulsive jet depends on the ACW strength and the gastric fluid properties (Pal et al., 2004; Alokaily et al., 2019). In the present study, the peak jet velocity is about 3 cm/s. The velocity contours on the cross section at the body region are also shown in Figure 4B the retropulsive flow toward the fundus is observed here. However, the overall velocity magnitude is O(10^–3^) cm/s, which is very weak compared to the flow in the antrum region.

**FIGURE 5:**
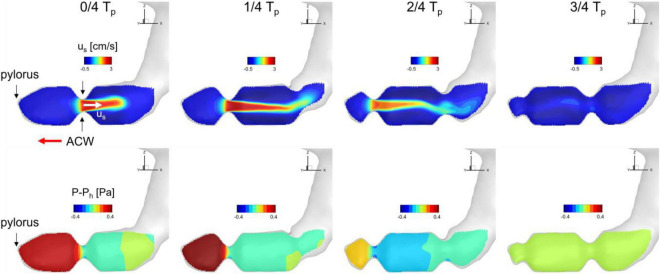
Snapshots of velocity and relative pressure contours on the antrum cross section. u_*s*_: velocity component along the antrum pointing away from the pylorus. P_*h*_: hydrostatic pressure.

To investigate the time-dependent flow pattern in the antrum region and the development of the retropulsive jet, the snapshots of the flow velocity and relative pressure on the antrum cross-sectional plane (see Figure 2B) are plotted in Figure 5. At 0/4 T_*p*_ the ACW is propagating toward the pylorus and this traveling contraction with the closed pylorus increases the pressure downstream. The pressure drop across the ACW develops the retropulsive jet. As the ACW is propagating closer to the pylorus the downstream pressure is further increased and the retropulsive jet becomes stronger (1/4 T_*p*_). The ACW is then diminishing as it reaches the pylorus, and this releases the downstream pressure and also weakens the jet (2/4 T_*p*_). When the ACW reaches the pylorus the retropulsive jet disappears and no pressure drop across the antrum is observed (3/4 T_*p*_). The process is repeated as the next ACW is coming and this generates the pulses of the jet (i.e., retropulsive jet). Since the retropulsive jet makes the strong and energetic flows in the antrum region, it should have a significant impact on the pill motion and drug dissolution.

### Pill Motion and Dissolution

In order to investigate the pill interacting with the gastric flow and its dissolution in the stomach, fluid-structure interaction simulations are performed employing the pill model described above. The simulation for one ACW cycle (20 s) took about 138,240 CPU h (240 h wall-clock time with 576 CPU cores) on the TACC Stampede 2 cluster.^1^ In this study, we considered two different pill densities to study the effects of gravity as well. It is also important to note that the pill density can be modulated in the oral drug design to control the residence time of the pill in the GI system (Gupta et al., 2009). The first case, the specific gravity (SG) of the pill is set to 1 so that the pill is neutrally buoyant and the gravity does not play a role on the pill motion, and, for the second case, SG is 1.2, which is the actual specific gravity of salicylic acid pill (Bai and Armenante, 2009). The pill is initially placed at the entrance to the antrum region (see Figure 2A) and the simulations are performed for 60 s (3 ACW cycles).

**FIGURE 6:**
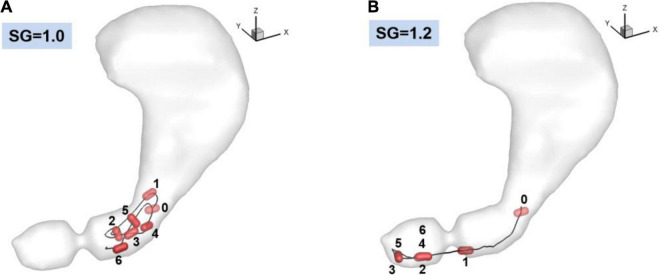
Trajectories of the pill in the stomach. 0: Initial position. 1–6: Positions at every 10 s. **(A)** SG = 1.0 (neutrally buoyant pill), **(B)** SG = 1.2.

The trajectories of the pill for 60 s obtained from the fluid-structure interaction simulations are plotted in Figure 6 for SG = 1.0 and 1.2. For the neutrally buoyant case (SG = 1.0), the pill floats around the entrance region of the antrum due to its interaction with the gastric flow. It is observed that the pill is always located upstream of the ACW because it continues to be pushed out by the retropulsive jet. The heavier pill (SG = 1.2), on the other hand, quickly settles down by the action of gravity (Figure 6B, 0–1), and displaces out of the core of the retropulsive jet. The pill itself is therefore not pushed out by the retropulsive jet but is pushed toward the pylorus directly by the antral wall contraction (Figure 6B, 1–3). Once the wall contraction is released, it is again settled down by the gravity (Figure 6B, 3–4). In this way, the pill is always located downstream of the ACW and near the pylorus.

**FIGURE 7:**
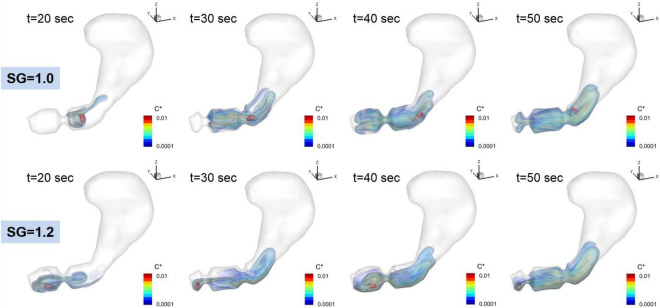
Time dependent volumetric distributions of the dissolved API concentrations. C* = *C*_*API*_/*C*_*s*_ is the normalized concentration.

**FIGURE 8:**
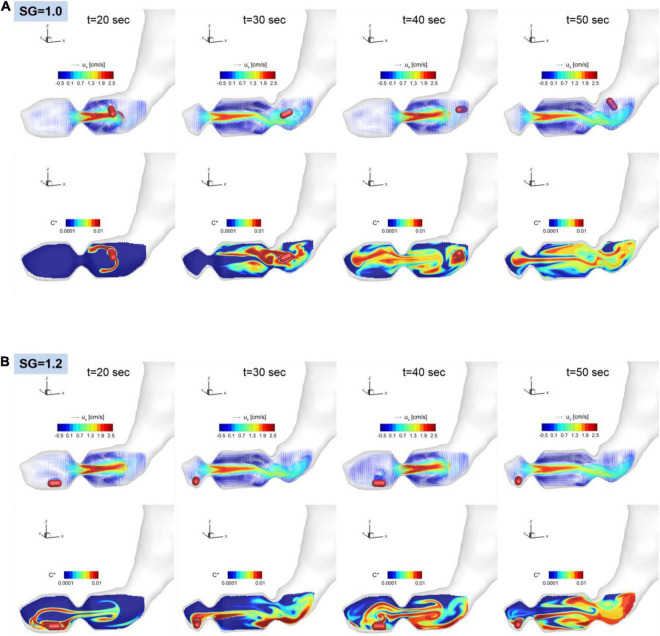
Snapshots of the velocity vectors and API concentration contours on the antrum cross-sectional plane for SG = 1.0 **(A)** and 1.2 **(B)**.

The dissolution of the pill (surface erosion) is simulated by directly solving the convection and diffusion equation for the API concentration (Eq. 11) for the duration of 60 s, and the volumetric distributions of dissolved API concentration are shown in Figure 7 for SG = 1.0 and 1.2. Further details of the interactions between the gastric flow and the transport of API concentration are shown in Figure 8 for SG = 1.0 and 1.2. These figures show the velocity vectors and API concentration contours on the antrum cross-sectional plane.

For SG = 1.0, the pill is located superior to the ACW and dissolved there. The dissolved API is, however, entrained by the recirculating flow around the retropulsive jet and transported into the antrum. At the same time, the API is also transported toward the body of the stomach by the retropulsive jet. As a result, the API is distributed over the entire antrum except very near the pylorus. For SG = 1.2, the pill is located very near to the pylorus. The dissolved API concentration is then transported by the retropulsive jet to a region superior to the ACW and toward the body. The API is then mixed in the antrum by the retropulsive jet and the re-circulating flow. It is interesting to note that although the locations of the pill are very different for SG = 1.0 and 1.2, after a while (*t* = 50 s), the overall distributions of the API are very similar for both cases except very near the pylorus. This is because of high quality mixing driven by the ACW and the retropulsive jet.

### Mass Transfer Coefficient

The dissolution rate or mass transfer coefficient is one important metric to quantify drug dissolution (Bai and Armenante, 2009). For a non-disintegrating drug, the dissolution occurs primarily by the surface erosion. A well-known model of the dissolution is the Nernst-Brunner equation (Nernst, 1904):


(14) $$\frac{d\,m_{d}}{d\,t}=A_{p}\,\frac{D}{\delta_{d}}\,\left(C_{s}-C_{\infty }\right)=k_{m}\,A_{p}\,\left(C_{s}-C_{\infty }\right),$$

where *m*_*d*_ is the dissolved mass, *A*_*p*_ is the surface area of the drug, *D* is the diffusion coefficient, *δ_*d*_* is the apparent diffusion boundary layer thickness, *C*_*s*_ is the solubility, *C_∞_ is the bulk concentration in the medium, and *k_*m*_* = *D*/*δ_*d*_* is the mass transfer coefficient. The bulk concentration can be estimated by *C*_∞_ = m_d_/V*, where _*V*_is total volume of the medium. At the early stage or for the case where the volume of the medium is much larger compared to the pill, *C*_∞_ is much smaller than *C*_*s*_ and thus negligible, and the solution of (Eq. 14) yields the linear profile of the dissolved mass (Healy et al., 2002):


(15) $$m_{d}=k_{m}\,A_{p}\,C_{s}\,t.$$

At the later stage or for the case where the volume of the medium is small, *C_∞_ = m_d_/V* has to be included and the solution exhibits an exponential profile.

**FIGURE 9:**
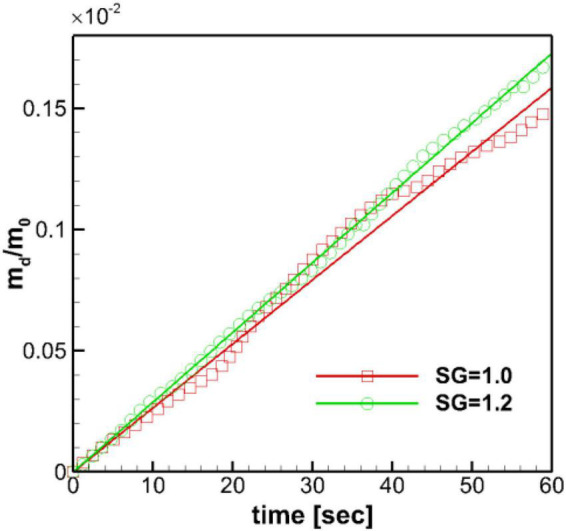
Time profiles of the normalized dissolved mass of the pill in the stomach. m_0_: initial pill mass. Solid lines: best-fit linear profiles.

In the present study, the dissolved mass in the stomach is monitored in time, and the profiles of the normalized dissolved mass are plotted in Figure 9 for both SG = 1.0 and 1.2. For the duration of initial 60 s, the time profiles of the dissolved mass follow the linear function (Eq. 15) as expected. The heavier pill (SG = 1.2) follows the linear trend better, while the neutrally buoyant pill (SG = 1.0) shows slight oscillatory deviations, of which the period is close to the ACW cycle (∼ 20 s). The mass transfer coefficient, *k*_*m*_, can be evaluated from the slope of the best-fit line. The evaluated mass transfer coefficient is about 0.0011 cm/s for SG = 1.0 and 0.0012 cm/s for SG = 1.2. These values are apparently smaller than the ones measured by Bai and Armenante (2009) in the experiments with the USP-II device (*k_*m*_* = 0.005–0.01 cm/s) (Bai and Armenante, 2009).

### Wall Shear Rate and Mass Transfer

The mass transfer from the surface of the pill is governed by mass diffusion, and the mass transfer rate is directly proportional to the wall normal gradient of the concentration (Eq. 12). The fluid flow over the pill surface can increase the concentration gradient and thus, the mass transfer rate. The strength of the fluid flow over the surface is related to the wall shear stress or wall shear rate, and this suggests that the concentration gradient may be correlated to the wall shear rate. We can rationalize this by considering the diffusive mass flux to be analogous to the diffusive momentum flux (shear stress) from the classical Reynolds analogy in heat transfer (Geankoplis, 2003). In order to understand the effects of gastric flow on the mass transfer rate further, we investigated the correlation between the wall shear rate and the concentration gradient on the surface of the pill for both SG = 1.0 and 1.2.

**FIGURE 10:**
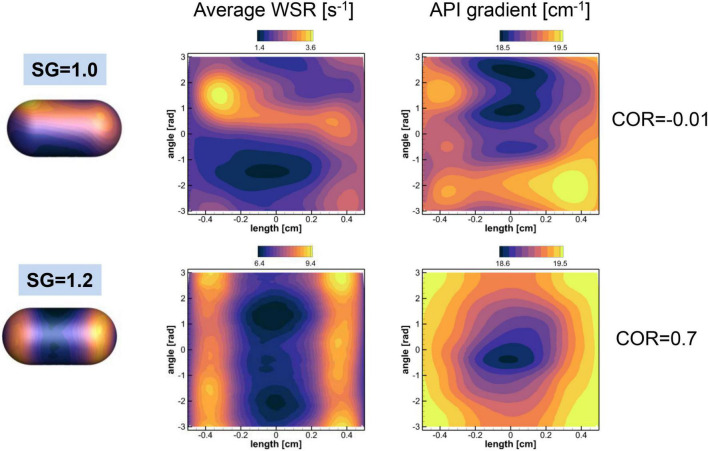
Distributions of the time averaged wall shear rate (WSR) and normalized API concentration gradient on the pill surface. The contour ranges are set by the min-max values of the variables. The spatial correlations between two quantities are also shown.

The wall shear rate magnitude, WSR, and the normalized API concentration gradient, CG, are calculated on the pill surface by


(16) $$\text{WSR}=\left|\frac{\partial \vec{u}}{\partial n}\right|,\text{CG}=-\frac{1}{C_{s}}\,\frac{\partial C}{\partial n},$$

where *n* denotes the direction normal to the pill surface. Note that the wall shear stress magnitude is given by μ × (WSR) and the local mass flux is given by *DC*_*s*_.(CG). These quantities are averaged over time in a Lagrangian manner for the duration of the pill motion shown in Figure 6. The spatial distributions of the time averaged WSR and CG are plotted in Figure 10 for SG = 1.0 and 1.2. The average WSR for SG = 1.0 is higher on one side than the other. The overall average value (time and space) is 2.19 s^–1^. For SG = 1.2, the overall average value of the WSR is 7.65 s^–1^, which is noticeably higher than SG = 1.0. This indicates that the relative flow strength over the pill is stronger for SG = 1.2 than SG = 1.0. It is because the neutrally buoyant pill (SG = 1.0) is convected by the flow, and thus the relative velocity between the pill surface and the flow is small. The average WSR and CG for SG = 1.2 show the similar pattern: higher values at the sides and lower in the middle. Overall average value of CG for SG = 1.2 is 19.2 cm^–1^ which is slightly higher than one for SG = 1.0, 19.0 cm^–1^, and this explains the slightly higher mass transfer coefficient for SG = 1.2.

To check the local correlation between the WSR and CG on the pill surface, the spatial correlation coefficient between two quantities is computed by


(17) $$\text{COR}=\frac{\bar{\phi_{1}^{\prime }\cdot \phi_{2}^{\prime }}}{\sqrt{\bar{{\left(\phi_{1}^{\prime }\right)}^{2}}\,\bar{{\left(\phi_{2}^{\prime }\right)}^{2}}}},\phi_{i}^{\prime }=\phi_{i}-{\bar{\phi }}_{i},$$

where bar denotes spatial average, ϕ_1_ = WSR, and ϕ_2_ = CG. For SG = 1.0, the COR is -0.01, which means that two quantities are not correlated. For SG = 1.2, however, the WSR and CG are correlated well with COR = 0.7. The results show that the WSR (or wall shear stress) and the concentration gradient (or mass flux) are locally correlated only when the relative flow strength over the pill is substantially larger.

### Dissolved Mass Near the Pylorus

The API is eventually meant to be absorbed in the intestine and the flux of the API through the pylorus into the duodenum determine the rate of availability of the API in the intestines. Although the emptying through the pylorus is not included in the current mode, the emptying rate can be estimated by the API concentration or dissolved mass in the pyloric region, since the emptying rate is proportional to the concentration at the pylorus.

**FIGURE 11:**
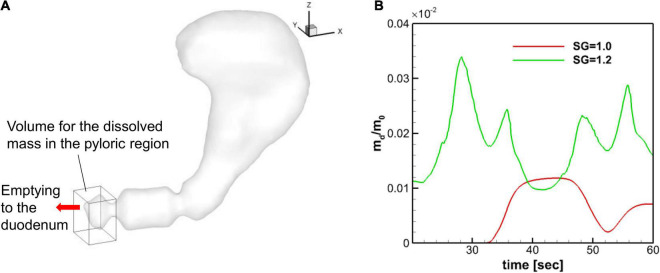
Dissolved mass in the pyloric region. **(A)** Volume used for the evaluation of the dissolved mass in the pyloric region. The size of the volume is 2 cm, 2.5 cm, and 3 cm in x, y, and z directions, respectively. **(B)** Time profiles of the normalized dissolved mass in the pyloric region.

The dissolved mass of the pill in the pyloric region is obtained by integrating the API concentration over the volume within 2 cm from the pylorus (see Figure 11A), and the time profiles of the dissolved mass in the pyloric region are plotted in Figure 11B for SG = 1.0 and 1.2. Overall, the amount of dissolved mass in the pyloric region is larger for the heavier pill (SG = 1.2), primarily because the pill stays near the pylorus. For the neutrally buoyant pill (SG = 1.0), the dissolved mass arrives at the pyloric region after about 30 s and the peak value is about 3 times smaller than the SG = 1.2 case. For both cases, the profiles fluctuate with the ACW period (∼20 s) because of the retropulsive jet. The result indicates that the heavier pill should have the faster drug emptying rate.

## Discussion

We investigated the interaction between the gastric flow and pill dissolution in a physiological model of the human stomach using a computational model. The motion of the pill and its dissolution pattern are studied in detail for two different pill densities. The gastric flow can be characterized by a strong retropulsive jet in the antrum generated by the antral contraction wave (ACW) and the recirculating flow around the jet as shown in previous studies (Pal et al., 2004; Ferrua and Singh, 2010; Ferrua et al., 2011). These characteristic gastric flows play an important role in the motion of the pill as well as the transport and mixing of the dissolved API. Gravitational force is another important factor affecting the pill motion, and for that reason we considered both a neutrally buoyant pill (SG = 1.0) and one slightly heavier than the medium (SG = 1.2). It is observed that the neutrally buoyant pill buffeted by the gastric flow. If the pill is initially placed superior to the ACW, it cannot enter the antrum because it is continually pushed back by the strong retropulsive jet. We observed, however, that the dissolved API can be transported into the antrum and the pyloric region by the recirculating flow around the jet. On the other hand, the heavier pill quickly settles down due to gravity, and the gastric flow plays little role in the pill motion. The pill is pushed toward the pylorus mainly by its interaction with the antrum wall and the ACW, and the pill stays in the downstream antrum region. The dissolved API is, however, transported toward the body of the stomach by the retropulsive jet. For both SG = 1.0 and 1.2, the gastric flow generated by the ACW effectively mixes the dissolved API in the antrum. We found that although the pill motions and the dissolution locations are very different for SG = 1.0 and 1.2, the overall distributions of the API quickly become very similar for both cases except very near the pylorus.

The overall dissolution rate of the pills is also studied by monitoring the time profiles of the dissolved mass. The linear profiles are observed for both pills as expected by the Nernst-Brunner equation. Although our simulations were performed for a relatively short time duration (∼ 60 s), the previous experimental studies also have shown a linear dissolution profile for the extended duration (> 40 min) (Healy et al., 2002; Abrahamsson et al., 2005; Bai and Armenante, 2009). The evaluated mass transfer coefficients for the pills with SG = 1.0 and 1.2 were not very different, with the SG = 1.2 case showing a slightly higher value. These values are, however, about 5–10 times smaller than the those measured in the experiments with the USP-II device (Bai and Armenante, 2009) for the same salicylic acid tablet. The primary reason for this discrepancy is the different flow strength between the physiological stomach model and the USP-II device. The strong fluid flow over the pill surface can result in forced mass convection and the higher local mass flux. The flow strength over the surface can be estimated by the wall shear rate or the wall shear stress. It has been reported that the wall shear rate on the pill surface in the USP-II device ranged about 80–180 s^–1^ depending on the pill location and the mass transfer coefficient was proportional to the wall shear rate (Bai and Armenante, 2009). In our physiological human stomach model, the wall shear rate on the pill surface was only about 7.65 s^–1^ for SG = 1.2 and 2.19 s^–1^ for SG = 1.0. This is because the flow velocity in the physiological stomach model is much lower than in the USP-II device. The peak retropulsive jet velocity was about 3 cm/s in our simulation, while the paddle tip velocity in the USP-II device was about 30 cm/s at 100 RPM (Bai and Armenante, 2009). Another reason for this difference is that the pills move along with the flow in the stomach, whereas in the USP-II device, that are fixed at one location.

To understand the relation between the flow strength and the dissolution mass flux, we investigated the correlation between the wall shear rate and the API concentration gradient. These quantities represent the relative flow strength over the pill and the local diffusive mass flux, respectively. We have found that if there is substantial relative flow motion over the pill surface, the wall shear rate and the mass flux are indeed correlated not only globally as shown in the previous studies (Abrahamsson et al., 2005; Bai and Armenante, 2009; Wang and Brasseur, 2019), but also locally as shown in the present study. It is observed, however, that if there is little relative motion between the pill and the flow, like the neutrally buoyant pill, the mass flux (concentration gradient) is almost independent of the wall shear rate. This suggest that the positive correlation between the wall shear rate and the mass flux may only be valid for the pills heavier than the medium. This may be an important finding because many dissolution models have been derived from the correlation between the mass flux and the wall shear stress or wall shear rate (Abrahamsson et al., 2005; Bai and Armenante, 2009; Wang and Brasseur, 2019).

Finally, we studied the effect of the pill density on the expected emptying rate of the API. If the drug is designed to be absorbed in the intestines, a faster emptying rate of the API into the duodenum through the pylorus could enhance the bioavailability of the API (Dressman et al., 1998). In the present study, the emptying through the pylorus was not modeled directly, so we used the dissolved mass in the pyloric region as a surrogate for the API emptying rate, since the emptying rate is proportional to the concentration at the pylorus. It is observed that the dissolved mass in the pyloric region for the heavier pill (SG = 1.2) is about 3 times higher than the neutrally buoyant pill (SG = 1.0). This is because the heavier pill stays near the pylorus, while the neutrally buoyant pill never enters the antrum, and the dissolved API is only transported by the recirculating flow. The result shows that the heavier pill can be more effective for the faster emptying of the API into the duodenum. It is important to note, however, that the settled-down location of the heavier pill strongly depends on the direction of the gravity and thus on the body posture. For example, if a person is lying down, the heavier pill could settle down in the middle of the stomach instead of near the pylorus, and it will result in a much slower emptying rate of the API. These effects of body posture on the drug dissolution and API availability in the duodenum would be very interesting research topics for the future studies that can be tackled with the *in-silico* stomach models. Another interesting subject is the effect of gastric contents that will change the density and viscosity of the gastric fluid.

The present drug dissolution simulations are performed for a limited time duration (∼ 60 s). Although the early stage simulation results presented here have shown the importance of the interaction between the gastric flow and the pill motion on the physiological drug dissolution process, simulations over longer duration [∼ O(10) min] may be necessary to study the whole dissolution process. This will require a computational technique to accelerate the simulation because the full fluid-structure-mass transport interaction simulation is computationally very costly. One possible solution is segregating the fluid-structure interaction and mass transport simulations, since they do have very different time scales. Another important thing to consider for the physiological drug dissolution simulation for a longer time duration is the gastric emptying through the pylorus. As discussed above, the API in the stomach will eventually empty into the duodenum. Thus, the API emptying through the pylorus needs to be included in the model. Drug dissolution modeling over longer time period will be pursued in future studies by employing a model for gastric emptying and a method to accelerate the simulation.

## Data Availability Statement

The raw data supporting the conclusions of this article will be made available by the authors, without undue reservation.

## Author Contributions

JHS and RM contributed to conception and design of the study. JHS performed the computational modeling and simulation, analyzed the results, and wrote the first draft of the manuscript. Both authors contributed to manuscript revision, read, and approved the submitted version.

## Conflict of Interest

The authors declare that the research was conducted in the absence of any commercial or financial relationships that could be construed as a potential conflict of interest.

## Publisher’s Note

All claims expressed in this article are solely those of the authors and do not necessarily represent those of their affiliated organizations, or those of the publisher, the editors and the reviewers. Any product that may be evaluated in this article, or claim that may be made by its manufacturer, is not guaranteed or endorsed by the publisher.

## Figures and Tables

**APPENDIX FIGURE A1:**
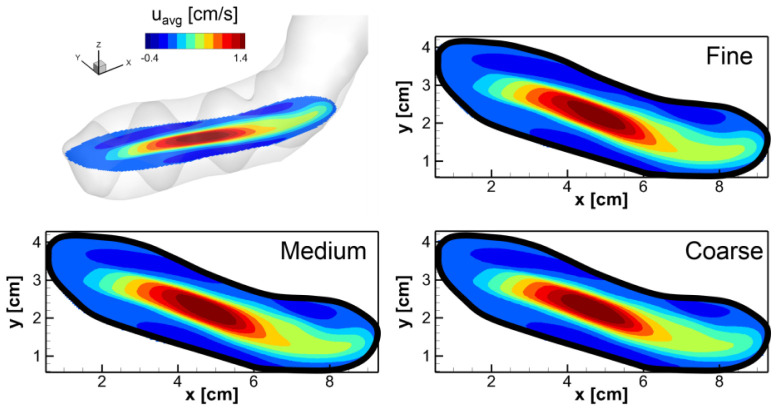
Comparison of the time averaged velocity fields on the horizontal cross-section in the antrum for three different grid resolutions: Coarse (225 × 100 × 150), Medium (360 × 160 × 240), and Fine (576 × 256 × 384). The velocity component in the x-direction (u) is averaged for one ACW cycle (20 s).

**APPENDIX FIGURE A2:**
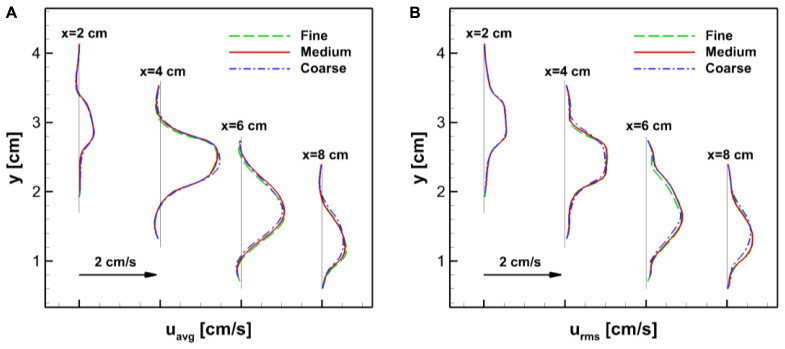
Comparisons of the velocity profiles at four different locations (x = 2,4,6, and 8 cm) on the horizontal cross-section in the antrum (see Appendix Figure A1). **(A)** Time averaged velocity in the x-direction. **(B)** Root-mean-squared velocity fluctuation.

## Footnotes

1
www.tacc.utexas.edu

## Appendix

### A. Grid Convergence Study

In order to assess the grid resolution used in the simulations, we tried uniform Cartesian grids with three different resolutions: Coarse (225 × 100 × 150), Medium (360 × 160 × 240, about 14 million points), and Fine (576 × 256 × 384, about 57 million points). The gastric flow simulations are performed for 3 ACW cycles with each grid and the velocity fields are compared. In Appendix Figure A1, the time averaged velocity fields for the x-direction component (u) are plotted for all three resolutions. The comparison is made on the horizontal cross-section in the antrum where the gastric flow is the most energetic. The figure shows that the averaged velocity fields are almost identical for all three resolutions. More quantitative comparisons are made in Appendix Figure A2, where the time average (u_avg_) as well as root-mean-squared fluctuating (u_rms_) velocity profiles are plotted at four different locations. Overall, the velocity profiles match well with each other. While there is a little discrepancy for the coarse resolution comparing to the medium and fine ones, the maximum difference between the medium and fine resolutions is found to be only 5% of the peak velocity magnitude. Based on this assessment, the medium resolution is used for all the simulations presented in this study.
